# Fatigue Life Assessment of the Shell Structure of Purified Terephthalic Acid Filter Press

**DOI:** 10.3390/ma13153276

**Published:** 2020-07-23

**Authors:** Ming Song, Weiya Zhang, Wenchun Jiang, Jinguang Wang, Xu Zhao, Xiangnan Zhai

**Affiliations:** 1 School of Pipeline and Civil Engineering, China University of Petroleum (East China), Qingdao 266580, China; songmingx@gmail.com; 2 State Key Laboratory of Safety and Control for Chemicals, SINOPEC Research Institute of Safety Engineering, Qingdao 266101, China; zhangweiya187@163.com; 3 School of New Energy, China University of Petroleum (East China), Qingdao 266580, China; 4 SINOPEC Engineering Incorporation, Beijing 100101, China; wangjinguang@sei.com.cn; 5 Tianhua Research Institute of Chemical Machinery and Automation, Lanzhou 730060, China; zhaoxu65@vip.163.com (X.Z.); zhaixiangnan@126.com (X.Z.)

**Keywords:** PTA filter press, fatigue life, failure probability, structure optimization

## Abstract

The filter press is one of the most important devices in purified terephthalic acid (PTA) refinement, and it is of great significance to ensure the fatigue strength of the structure in operation. In this study, the fatigue life prediction of the shell structure of the PTA filter press was investigated through numerical and experimental methods. Firstly, the accurate stress at the critical area of the stiffener was obtained based on the thermomechanical model and submodel approach proposed. Subsequently, the fatigue life was evaluated by the fatigue strength reduction method and hot spot stress method. Finally, the shell structure is optimized by increasing the size of the axial stiffener and continuous hoop stiffener. The results unveil that both thermal load and outer structure constraints have little effect on the radial displacement and stress amplitude of the shell structure. Through modifying the fatigue design curve of the fatigue strength reduction method, the shell structure of the PTA filter press has 42.0% and 0.3% failure probabilities in the previous and present cyclic pressure conditions. Furthermore, the hoop stiffener plays an important role in reducing radial displacement and stress amplitude, among which three hoop stiffeners exhibit the most satisfactory optimization.

## 1. Introduction

The purified terephthalic acid (PTA) is one of the most important chemical raw materials, which is widely used in the chemical, electronics and construction industries [1,2]. The filter press is the substitute of pressure centrifuge and vacuum filter [3], and the successful trial of the filter press in the PTA industry has led an improvement in the refinement unit and generated considerable benefits. The filter press has the function of filtrating, washing, and drying of the PTA and stands out for its advantages of simpler structure, shorter process, and lower costs of manufacturing, operating, and maintenance. However, the shell structure suffers pressure fluctuation as the slurry is fed discontinuously. After a long time service, the structure is inevitably subjected to fatigue damage, which makes it urgent to ensure the fatigue strength of the structure in operation. To enhance the stiffness of the shell structure, many reinforced stiffeners are welded to the external surface, but the shortcoming is that the existence of large number of weld joints makes it sensitive to failure [4]. Usually, the researchers mainly focus on the single weld joints in the laboratory [5], which is independent of the complex structure. However, the weld joints are exposed to more complicated conditions, e.g., the combination of weld residual stress [6,7], the thermal stress [8], the external loads [7,9], post-weld heat treatment [7], etc. Those studies are bound to show different fatigue failure mechanisms. To reveal the relationship between the actual complex welding structure and the simple welding sample, Gao et al. [10] studied the effect of constraint stress on the microstructure and properties of the electron beam welded joint and found that the constraint conditions lead to a large number of dislocations in the martensite and higher tensile strength. Schork et al. [11] concluded that the fatigue strength is closely related with the weld toe radius *ρ*, excess weld metal height *h*, and flank angle *α*. Harish et al. [12] studied the specimen configuration on the fatigue behavior and exhibited the difference between the tensile-shear spot welds and the coach-peel spot welds. Hence, great attention should be paid to the fatigue analysis of the specific complex structure instead of the simple sample. 

The fatigue design criterions have been widely used to assess the fatigue life of structures [13,14]. Obviously, the regulations governing these kinds of components must be strict enough to ensure the structural integrity of the pressure vessel. Joshi et al. [15] presented a comparative study on the application of several existing design criterions for the fatigue life prediction of a typical four-member dragline cluster in a BE 1370 boom, and they concluded that much thicker and heavier structures should be used, as the current design codes applicable to such welded structures provide very conservative results. Yapici et al. [16] analyzed the fatigue curves given in ASME Boiler and Pressure Vessel Code Section VIII Division 2 [17] and EN 13445 Unfired Pressure Vessels Part 3 Codes [18], and the results show that the ring flange joint type used in the dryer is not appropriate both in terms of design itself and weld dimensions. Moreover, the design codes that currently exist may give a varying and sometimes discordant estimation of fatigue life. Kalnins et al. [19] performed fatigue analysis for a full-penetration joint welded from both sides and compared the fatigue life predicted by the ASME Boiler and Pressure Vessel Code with the European Standard for unfired pressure vessels. They reported that for the same geometry and loading, the two codes would predict vastly different fatigue life. Therefore, researchers have devoted effects to modify the criterion standard. Yamada et al. [20] further reviewed conventional fatigue design procedures based on the maximum principal or normal stress range, and they proposed a more appropriate evaluation procedure. Dong et al. [21] introduced an equivalent structural stress parameter to investigate the non-proportional multi-axial fatigue of welded components, and the results show good consistency between the master stress versus the number of cycles to failure (S-N) curve adopted by the 2007 ASME Div. 2 and API 579 RP/ASME FFS-1 Codes and the consolidated S-N curve dominated by severe non-proportional multi-axial cyclic loading conditions. In this case, it is necessary to evaluate the fatigue strength of the structures with different criterions.

In this paper, the fatigue strength of the filter press is investigated. The specific cyclic pressure at the critical reinforced stiffener area is tested in the experimental procedure, and the fatigue analysis is performed based on the finite element method (FEM). Finally, a modification of the design code and optimization of the structure are proposed for the fatigue design of the PTA filter press.

## 2. Stress Fluctuation Measurement

**Figure 1:**
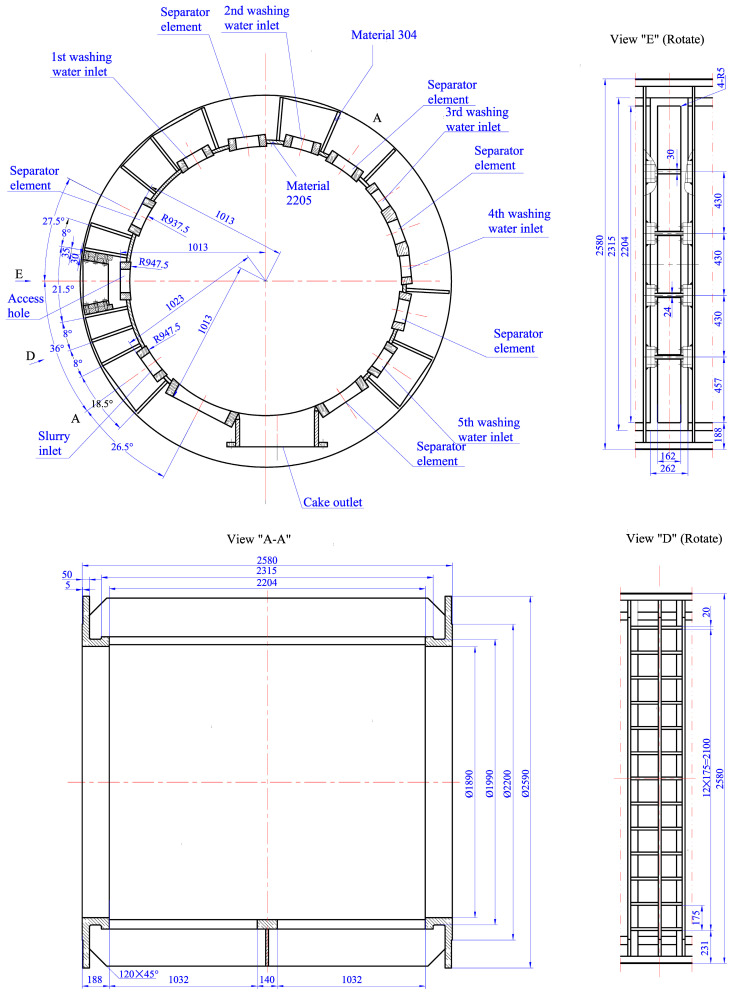
Specific dimensions of the working filter press (unit: mm).

**Figure 2:**
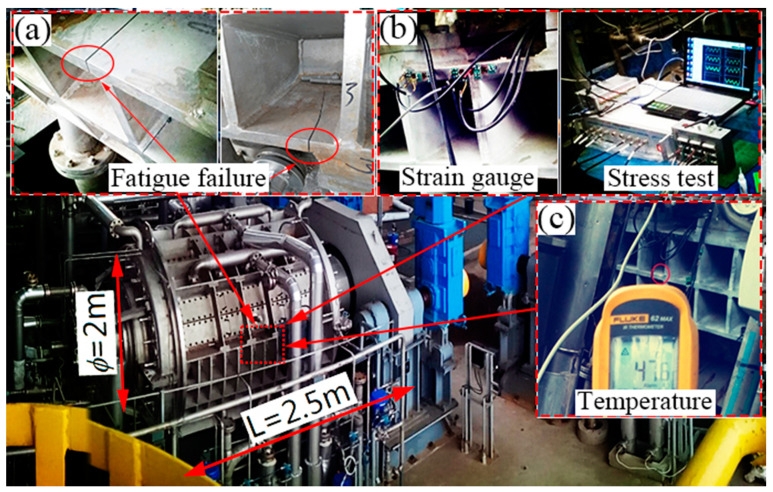
Purified terephthalic acid (PTA) solvent exchanger: (**a**) fatigue failure location, (**b**) fluctuating stress measurement, (**c**) temperature measurement.

**Figure 3:**
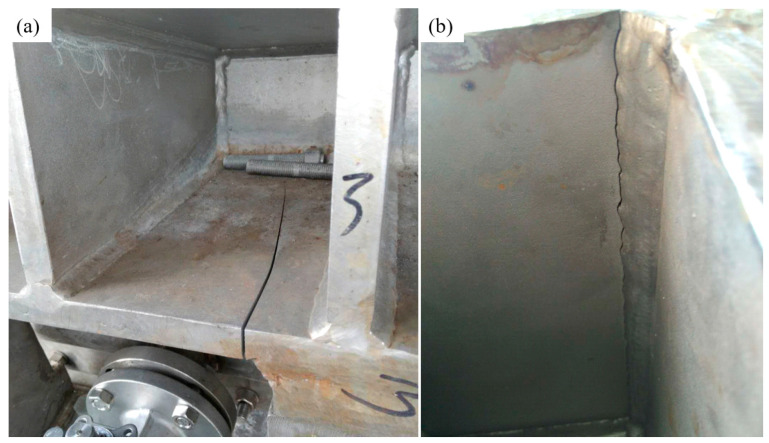
Fatigue cracks occurred at the weld toe: (**a**) the top surface and (**b**) the reverse side.

As shown in Figure 1, the basic procedures are as follows:

Firstly, under the pump pressure, the slurry fed into the filter press is squeezed on the filter screen. The solid filter cake is left on the filter screen, and the mother liquor is discharged from the outlet. Secondly, with the drum rotating, the filter cake is passed into the rough washing zone, and the washing liquid comes from the next stage. Thirdly, the filter cake is washed using the pure washing liquid. Fourthly, the compressed gas is passed into the filter press for drying, and the filter cake is blown out from the filter screen and unloaded. To perform different processes when the drum is rotating, the filter drum of the equipment is divided into numerous unit chambers, and the shell structure is divided into 4 to 5 mutually isolated and independent chambers by the inter-chamber seal. In this condition, when the slurry fed into the unit chamber discontinuously, the shell structure suffers a pressure fluctuation. The initial inner pressure ranges from 0.3 to 0.6 MPa with the frequency of 62 cycles/min, and the fatigue crack in a reinforced stiffener for a short life of only two months is observed (see Figure 2a and Figure 3). Renovation on the equipment is made to decrease the pressure fluctuation in the inner chamber, and the pressure ranges from 0.5 to 0.6 MPa with the frequency of 300 cycles/min.

To obtain the real fluctuating stress, the fluctuating stress measurement was made in the area located at the middle of the shell structure. The electrical measure method is used to measure the stress fluctuation in working condition, as shown in Figure 2b. The strains were measured by strain gauges. The workflow is as follows: Before the measurement, the solvent exchanger was shut down. The surface of the component was polished to the roughness *R*_a_ = 0.6, and then it was cleaned by acetone. After that, the strain gauge was pasted and solidified for over 4 hrs. Then, the soldering wires were connected with the strain gauge, HP-DY8125 dynamic stress–strain test analysis system and the computer. Finally, the on-site commissioning, as well as data recording and processing, were carried out. Meanwhile, to validate the thermal conduction process modeled by FEM, the temperature at the outer surface of the shell structure was measured using the non-contact infrared thermometer (see Figure 2c), and the temperature is 47.6 °C with the interior temperature of 105 °C of PTA.

## 3. Finite Element Analysis

### 3.1. Finite Element Model

**Figure 4:**
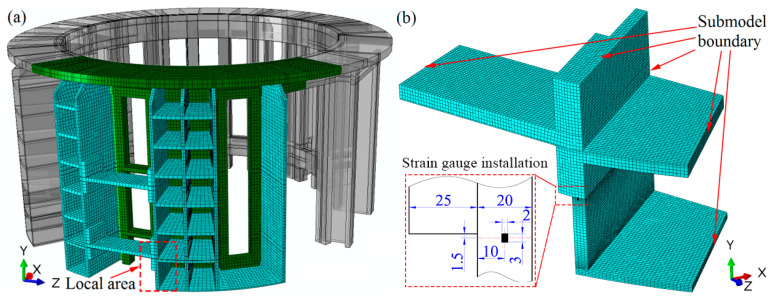
The meshing of the PTA filter press shell: (**a**) whole structure, (**b**) substructure, and illustration of strain gauge installation (unit: mm).

Due to the symmetry, an only one-quarter model of the filter press was built using the commercial software ABAQUS (6.10 version, ABAQUS Inc., Johnston, IA, USA), as shown in Figure 4a. The symmetric constraints were applied on the symmetric boundary. To get a higher resistance of corrosion, the stainless austenite-ferrite (SAF) 2205 was adopted to manufacture the shell structure, and the austenitic stainless steel (SS) 304 was used for the reinforced stiffener aiming to decrease the cost. The green region represents the SAF 2205 and the cyan region is for 304 SS, respectively. The number of elements and nodes are 28,715 and 44,736 for the global model, respectively.

Although the element number of the global model is large relative to the finite element model, the mesh density is rather coarse at the local area. To obtain an accurate result and save the computation time, a submodel approach was employed. Firstly, the displacement distribution of the whole model was calculated. After that, the local model was cut from the whole model, and the mesh was refined. The cutting boundary obtained from the global model was applied to the submodel. Figure 4b shows the mesh of the substructure model, and the number of elements and nodes are 43,224 and 53,525, respectively. The recalculated submodel can get a more accurate result in the local area.

### 3.2. Thermomechanical Coupled Modeling

As the temperature field of shell structure is inhomogeneous, the thermomechanical model was adopted, and the corresponding thermoelastic properties of SAF 2205 and 304 SS are listed in Table 1 [5]. The sequential coupling method was used for the thermal stress calculation. The thermal analysis was performed firstly, and then the mechanical analysis was implemented based on the thermal results. Specifically, the thermal analysis was implemented by adopting the 8-nodes thermal solid element of DC3D8R to acquire the temperature field in the thermal conduction and convection process. As the slurry fed into the chamber has the temperature of 105 °C, the interior surface was applied with the same temperature. The room temperature was set to 30 °C, and the natural convection coefficient is usually 5–25 W/(m^2^·k). In the present study, the natural convection coefficient was selected as 5 W/(m^2^·k) and applied to the outer surface. Later, the C3D8R element was replaced for the mechanical analysis with the thermal load acquired in the thermal analysis. After that, the fluctuating pressure was applied on the interior surface of the shell structure. The working conditions at the pressures of 0.6 MPa and 0.3 MPa were calculated for the old design equipment, while working conditions at the pressures of 0.6 MPa and 0.5 MPa were calculated for the new design equipment.

### 3.3. Criterions of Fatigue Life Evaluation

#### 3.3.1. Fatigue Strength Reduction Method of ASME VIII 2

The fatigue evaluation procedures of the ASME boiler and pressure vessel code, Section VIII 2 [17] are defined as follows:

Firstly, the amplitude of effective stress is calculated using the following equation:

(1) $$S_{a}=\frac{K_{f}\cdot K_{e}\cdot \Delta S}{2}$$

where *S*_a_ is the equivalent stress amplitude, *K*_f_ is the fatigue weaken coefficient considering the effect of notch and weld, and *K*_e_ is the fatigue loss coefficient, which is taken as 1 for pure elastic analysis. Δ*S* is the equivalent stress range, and the formula is defined as follows:

(2) $$\Delta S=\frac{1}{\sqrt{2}}{\left[\begin{array}{l} {\left(\Delta \sigma_{11,k}-\Delta \sigma_{22,k}\right)}^{2}+\\ {\left(\Delta \sigma_{11,k}-\Delta \sigma_{33,k}\right)}^{2}+\\ {\left(\Delta \sigma_{22,k}-\Delta \sigma_{33,k}\right)}^{2}\\ +6\left(\begin{array}{l} \Delta \sigma_{12,k}^{2}+\Delta \sigma_{13,k}^{2}+\\ \Delta \sigma_{23,k}^{2} \end{array}\right) \end{array}\right]}^{0.5}$$

The correction of effective stress amplitude for different materials is defined as:

(3) $$S_{a}^{\prime }=S_{a}\cdot \left(\frac{E_{\mathrm{FC}}}{E_{T}}\right)$$

*E*_FC_ is the elastic modulus used for the fatigue design curve, and the predefined value is 195 GPa. *E*_T_ represents the specific material’s elastic modulus at a certain temperature. 

The fatigue life can be obtained by the design fatigue curve with the definite stress amplitude *S*′_a_.

A fatigue strength reduction factor of 2.5 is recommended for the only visual examination of the surface. Therefore, by comparing the calculated equivalent stress amplitude with the fatigue design curve, the fatigue life is obtained. Note that the fatigue data shown in Table 3-F.3 [17] for the series 3XX high-alloy steels are used. The fatigue design curve came from the smooth round bar fatigue test and has considered the data scatter as well as the effect of size and environment, and it is modified by the mean stress.

#### 3.3.2. Hot Spot Stress Method of EN 13445-3

**Figure 5:**
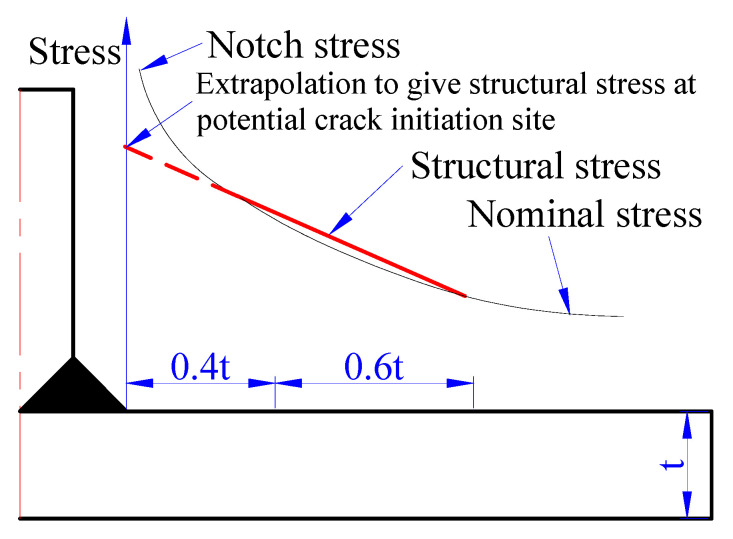
Extrapolation to obtain structural stress at a potential crack initiation site.

The rules for fatigue are compiled in Chapters 17 and 18 of EN 13445-3 (Unfired pressure vessels-Part 3: Design) [18]. The evaluation procedures for fatigue design in standard EN 13445-3 include the structural stresses concentration in the place using extrapolative techniques, the calculation of correction factors, the class for a certain weld joint type, and the characterization of fatigue (Whöler curve). The standard EN 13445-3 provides three techniques for determining the structural stresses in the heat-affected zone, and the linear extrapolation is used for component bending slightly. Figure 5 shows the distribution of nominal, structural, and notch stress at a structural discontinuity, and extrapolation to obtain structural stress at a potential crack initiation site.

The Tresca criterion is used for the equivalent stress in the multi-axial state, and the von Mises equivalent stress is also permitted. For welded joints, the correction factors are dependent on the joint thickness and temperature. The thickness-dependent factor *f*_t_ is equal to 1 when the thickness is less than 25 mm. The temperature-dependent factor *f*_T_ is equal to 1 and 0.97 when the temperature is 20 °C and 150 °C, respectively. The method of interpolation is used for the temperature in the range of 20 to 150 °C.

The standard EN 13445-3 includes 10 weld classes (from 32 to 100), and the classes define the fatigue strength of welded joints in the function of the number cycles. According to Table 18-4 [18], the class of weld details for the characterization of weld joint type is defined. For weld joints, the fatigue life curves are described by the following equations:

(4) $$\Delta \sigma =\left\{ \begin{array}{l} {\left(\frac{C_{1}}{N}\right)}^{\frac{1}{3}}\;\mathrm{for}\;N\leq 5\times 10^{6}\;\mathrm{cycles}\\ {\left(\frac{C_{2}}{N}\right)}^{\frac{1}{5}}\;\mathrm{for}\;N\geq 5\times 10^{6}\;\mathrm{cycles} \end{array}\right.$$

where Δ*σ* is the stress range, and *C*_1_, *C*_2_, are constants given in Table 17-2 [18]. 

According to the class of weld details for use with the structural equivalent stress range for branch connections in Table 18-4 [18], the crack is located at the weld toe in branch, the weld toe is dressed, and the welding class is defined as 80. The corresponding constants of fatigue curves are 1.02 × 10^12^ for *C*_1_ and 1.96 × 10^15^ for *C*_2_. The endurance limit (at *N* = 5 × 10^6^) is 58.9 MPa, and the cut-off limit (at *N* = 1 × 10^8^) is 32.4 MPa. Note that the notch effects of welds and the maximum possible influence of residual stresses have been taken into account in preparing the fatigue design curves.

## 4. Results and Discussion

### 4.1. Thermomechanical Stress Evaluation

**Figure 6:**
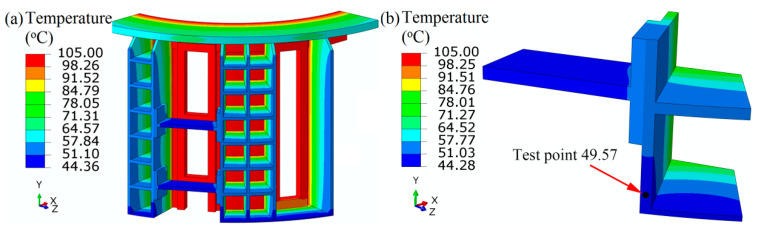
Temperature distribution of the whole PTA filter press (**a**) and the local (**b**).

Figure 6 shows the non-uniform temperature distribution of the filter press. Through heat conduction and convection, the temperature decreases from the inner surface to the outer surface (see Figure 6a). To validate the finite element analysis, the temperature at the outer shell is tested by the infrared thermometer. The temperature at the corresponding point of the stiffener is 49.57 °C, which is almost consistent with the experimental value 47.60 °C (see Figure 6b). The non-uniform temperature distribution is bound to affect the temperature-dependent material properties and strength.

**Figure 7:**
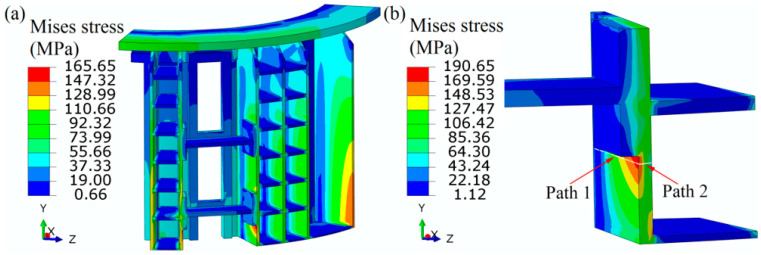
Von Mises stress distributions of the whole PTA filter press (**a**) and local area (**b**).

Figure 7 shows the combination of thermal stress and mechanical stress at the peak pressure of 0.6 MPa. The maximum von Mises is mainly located at the middle of axial stiffener, which is consistent with the failure location (see Figure 7a). As the mesh is coarse at the critical area, enough fine mesh is adopted in the submodel, and the submodel shows a higher maximum von Mises stress at the local area with the magnitude 190.65 MPa (see Figure 7b). The difference in maximum magnitude between the whole model and submodel is closely related to the mesh size, and the mesh size sensitivity is also researched in the next graph. To satisfy the strength by design, the stress evaluation is conducted. The allowable stress *S*_m_ is 140 MPa for 304 SS with the yield strength 210 MPa and the safety factor taken as 1.5. Obviously, the maximum von Mises stress is larger than the allowable stress and needs evaluation by design analysis. The stresses are split into membrane and bending stresses from the thermal stresses and geometric notch, and the linearization is necessary. The paths from the maximum to minimum values are shown in Figure 7b, and the linearized membrane *P*_m_ and bending *P*_b_ stresses are computed and listed in Table 2.

The average local membrane stresses are 118.63 MPa and 115.80 MPa for path 1 and path 2, which are named as the primary stresses and less than the allowable stress 1.5 *S*_m_. The maximum membrane plus bending stress is 221.09 MPa and 178.13 MPa for path 1 and path 2, which are named as the combination of primary and secondary stresses and are less than the allowable stress 3 *S*_m_. In this view, the static design analysis of the shell structure of the filter press is satisfied. 

### 4.2. Fatigue Life Validation and Prediction

**Figure 8:**
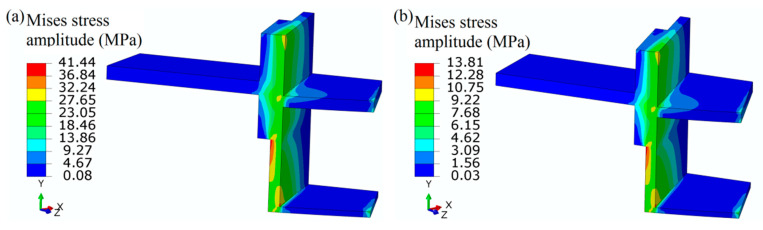
von-Mises stress amplitude at the cyclic pressure condition of 0.3 to 0.6 MPa (**a**) and 0.5 to 0.6 MPa (**b**).

**Figure 9:**
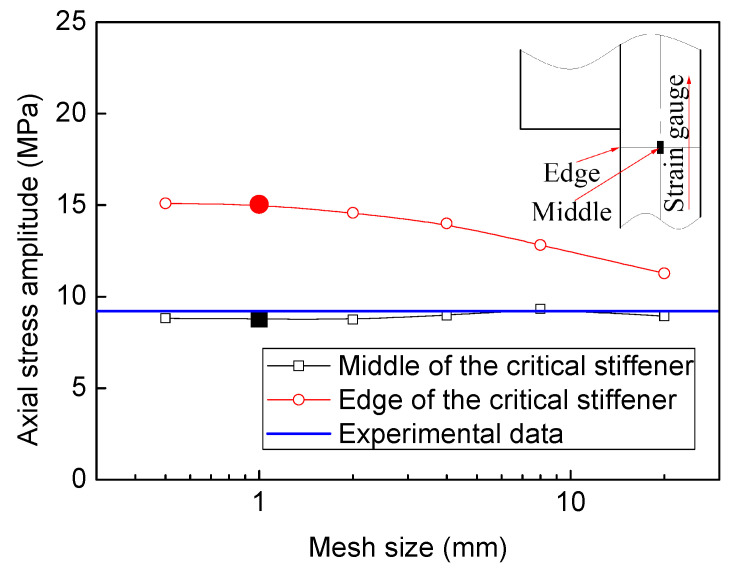
Effect of mesh size on the axial stress amplitude.

**Figure 10:**
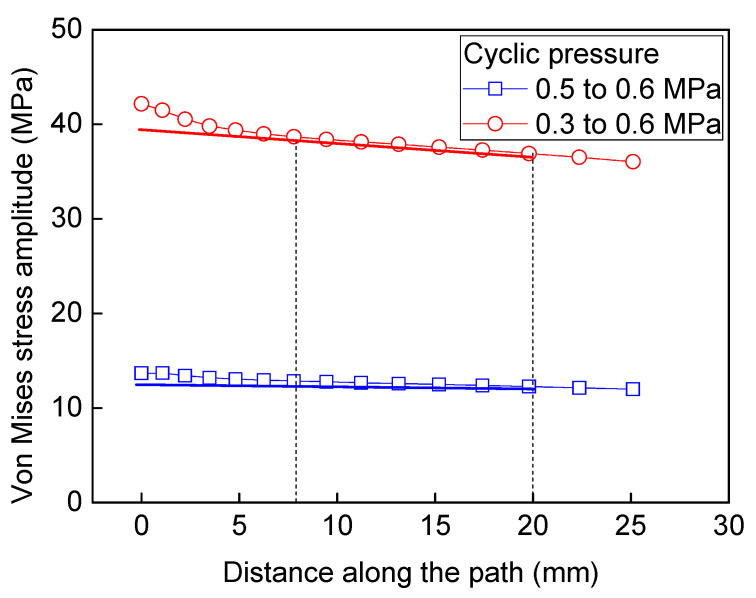
Extrapolation to obtain structural stress at potential crack initiation site at two different cyclic pressure conditions.

To enhance the stiffness, both hoop and axial stiffener are welded to the shell structure. From the results calculated by FEM, it is obvious that the stress distribution of the axial stiffener along the width is not uniform. Considering that the state of the stress ought to be multi-axial, the von Mises stress amplitude is used to evaluate the stress fluctuating at the critical area. The previous operation condition is that the cyclic pressure ranges from 0.6 to 0.3 MPa, and the calculated maximum von Mises equivalent stress amplitude is 41.44 MPa (see Figure 8a). In the present operating condition, the cyclic pressure ranges from 0.6 to 0.5 MPa, while the maximum Mises equivalent stress amplitude is 13.81 MPa at the critical region (see Figure 8b). In order to validate the stress fluctuation by FEM, the experiment was conducted. Considering the distribution and accuracy of the strain gauge, only the axial stress is validated. At the middle position of the stiffener corresponding with the strain gauge, the axial stress amplitude is 8.97 MPa. The calculated axial stress range by FEM is rather close to the experimental value 9.20 MPa, which demonstrates the validation of FEM. However, the maximum axial stress amplitude value is located at the edge of stiffener with the magnitude of 15.04 MPa. Although the submodel approach is adopted to increase the computation efficiency, the axial stress range at the local area is still dependent on mesh size. The mesh sensitivity analysis is carried out, as shown in Figure 9. As the mesh size decreases from 20 to 0.5 mm, the axial stress amplitude at the middle position of the critical stiffener is almost unchanged. When the mesh size decreases from 20 to 4 mm, the axial stress amplitude at the edge of the stiffener increases from 11.29 to 14 MPa linearly. As the mesh size decreases from 2 to 0.5 mm exponentially, the axial stress range at the edge of the stiffener increases slowly from 14.59 to 15.35 MPa. Therefore, the mesh size adopted as 1 mm × 1 mm in the submodel is capable of ensuring the computation efficiency and accuracy. Figure 10 shows the extrapolation to obtain the structural stress amplitude at a potential crack initiation site at two different cyclic pressure conditions. By extrapolation, the cyclic stress amplitudes are obtained at the critical positions with the magnitudes of 39.85 MPa and 13.28 MPa, respectively.

**Figure 11:**
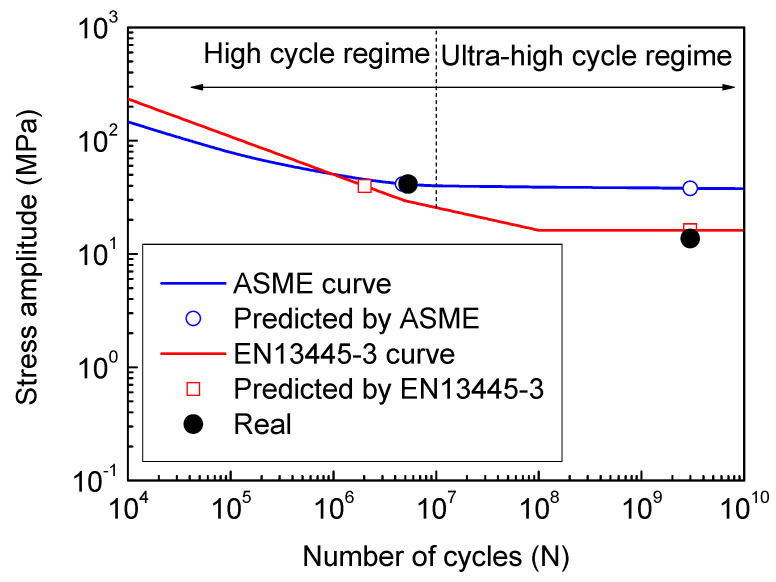
Fatigue life evaluation at two different cyclic pressure conditions.

In the previous working condition, the fatigue life of the shell structure belongs to the high cycle regime. Based on the data collected form the PTA filter presses with the same design, the corresponding average fatigue life is about 4.71 × 10^6^ cycles based on the fatigue strength reduction method (see Figure 11), which is a little less than the 5.36 × 10^6^ cycles (2 months), but rather close to the real fatigue life. Using the hot spot stress method, the corresponding fatigue life is 2.02 × 10^6^ cycles, which is less than the real fatigue life as well. Both the design methods give conservative prediction results, but they are the same order of magnitude. In this view, the fatigue life predicted by the two design methods is credible to some extent.

### 4.3. Discussion

#### 4.3.1. Working Condition on the Deformation and Stress Fluctuation

**Figure 12:**
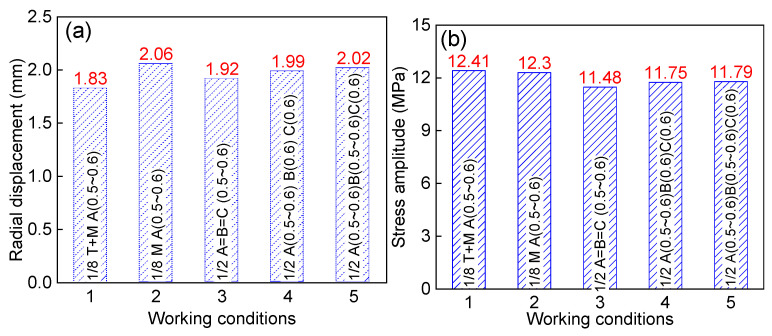
Effect of working condition on the shell structure: (**a**) radial displacement and (**b**) stress fluctuation amplitude.

In operation, the PTA filter press suffers from not only the mechanical stress but also the thermal load. Furthermore, in the structure analysis, the effect of the outer constraints on the concerned area should be considered carefully. Figure 12 shows the effect of working condition on the radial displacement and stress fluctuation amplitude. Without considering the thermal load (Condition 2), the radial displacement (2.06 mm) is a little larger than that (1.83 mm) considering the thermal load (Condition 1). However, the stress fluctuation amplitude shows little difference with the magnitude 12.3 MPa compared with 12.41 MPa. Strictly, the shell structure is unsymmetrical in structure and mechanical load. The half models (Condition 3, 4 and 5) were built to investigate the effect of structure and mechanical load. The concerned area is located at the region A, i.e., the original one-eighth model, and the other regions are divided into regions B and C equivalently. The regions of B and C suffer fluctuating pressures sometimes. The results show that the maximum radial displacements in the half models are a little larger than that of the one-eighth model, with the magnitudes of 1.92, 1.99, and 2.02 mm, respectively. However, the stress fluctuating amplitudes are a little smaller, with magnitudes of 11.48, 11.75, and 11.79 MPa, respectively. In summary, the thermal load and outer structure constraints have little effect on the radial displacement and stress amplitude, and the fluctuating pressure at the one-eighth model is the main factor.

#### 4.3.2. Comparison of the Fatigue Design Criterions

**Figure 13:**
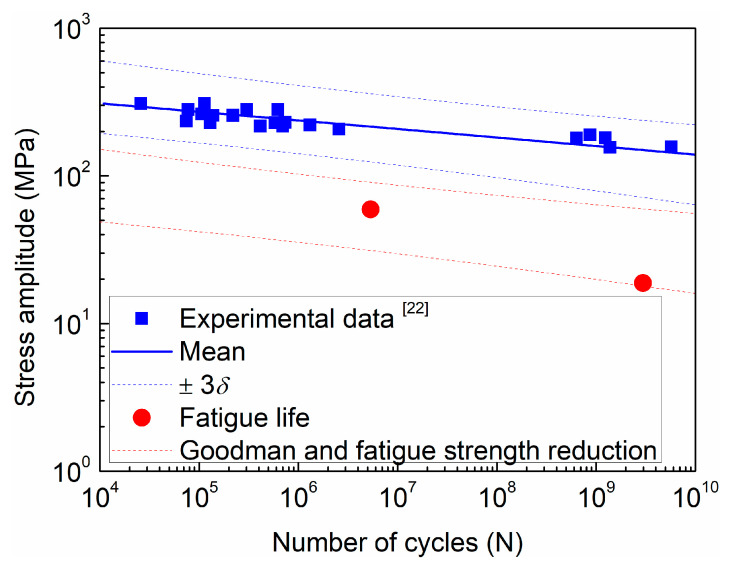
Fatigue life prediction by the modification of the fatigue design curve.

In the present working condition, the design fatigue life (2.98 × 10^9^) belongs to the ultra-high cycle regime. The maximum equivalent stress amplitude is 13.81 MPa, which is rather less than the stress amplitude 36.00 MPa at the design fatigue life given by the fatigue strength reduction method. The hot spot stress method recommends that the stress amplitude below the cut-off limit (16.20 MPa), i.e., the fatigue life exceeds 1 × 10^8^, is assumed to be non-damaging in fatigue, and need not be considered. The equivalent stress amplitude by extrapolation is 13.28 MPa, which is less than the cut-off limit and can get an infinite fatigue life. However, industry components demand a longer service life nowadays. There is growing attention on the ultra-high cycle fatigue (≥10^7^ cycles) behavior of metallic materials. A number of components and structures of vehicles, bridges, railways and aircraft are expected to exhibit cyclic loading greater than 10^8^ cycles [22,23]. Furthermore, there is a controversy as the weld joint shows no classical fatigue limit [24,25], and the experimental data of the 304 SS obtained by fatigue tests with a stress ratio of −1 and smooth specimens still show a stress-related trend in the ultra-high cycle regime [26], as shown in Figure 13. It is necessary to replace the universal design curve of the fatigue strength reduction method with a certain material S-N curve to obtain a more reasonable prediction of the shell structure of the PTA filter press. 

The data of the fatigue life of 304 SS at a given stress amplitude are shown in Figure 13, and the Weibull distribution [27,28] is one of the statistical models often used to represent the skewed distributions. The failure probability is calculated by the following equation:

(5) $$P=\frac{1}{\delta \sqrt{2\pi }}\int_{-\infty }^{\sigma }\exp\left[-\frac{1}{2}{\left(\frac{\sigma -\sigma_{m}}{\delta }\right)}^{2}\right]d\sigma .$$

For fatigue design, the BS ISO 12107 [29] was applied for the S-N statistical analysis. For a normal distribution, the S-N results for a 0.1% and 99.9% failure probability, i.e., three times the standard deviation (±3*δ*) of the logarithm of the fatigue life from the mean S-N curve for the population, at a 95% confidence level are adopted.

Considering the effect of weld residual stress and the mechanical mean stress, the Goodman equation is used, as shown in the following:

(6) $$\frac{\sigma_{a}}{\sigma_{a\left(R=-1\right)}}+\frac{\sigma_{m}}{\sigma_{b}}=1$$

where *σ*_a_ is the stress amplitude, *σ*_a(*R*=−1)_ is the stress amplitude with a stress ratio *R* equal to −1, *σ*_m_ is the mean stress, and *σ*_b_ is the ultimate tensile strength. At the heat-affected zone, i.e., the potential fatigue crack initiation site, the magnitude of residual stress is close to the yield strength [30]. Therefore, the mean stress is taken as the combination of the yield strength and the mean stress of the elastic stress analysis. For 304 SS, the yield strength and ultimate tensile strength are taken as 210 MPa and 564 MPa, respectively.

At the previous cyclic pressure condition of 0.3 to 0.6 MPa, the maximum equivalent stress amplitude is 59.28 MPa, which is located at the three times of the standard deviation (31.28 to 91.04 MPa), and the failure probability is 42%. At the present cyclic pressure condition of 0.5 to 0.6 MPa, the equivalent stress amplitude is 18.81 MPa, which is close to the lower limit (17.52 MPa) of the fatigue life, and the failure probability is 0.3%. In this case, the further improvement of the equipment should be made to ensure the fatigue strength in the design life.

#### 4.3.3. Mechanism of the Structure Deformation and Optimization

**Figure 14:**
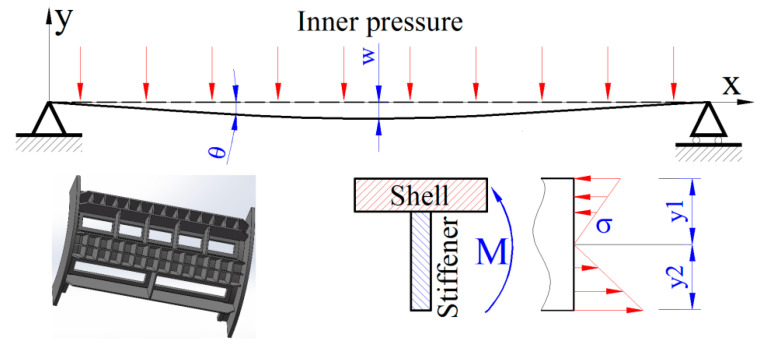
Loading simplification of the shell structure.

The stiffener aims to enhance the structural stiffness. If the deformation of the shell structure exceeds the critical value, the sealing effect between the drum and shell structure decreases dramatically, finally reducing the capacity. The slurry holes in the shell structure lead to the failure of structural integrity, and the part of the structure can be simplified as a T-shaped beam suffering from interior pressure [31,32], as shown in Figure 14. The stiffness of the beam is dependent on the material property, section, and the length [33]. However, the effect is not obvious by replacing the existing material, as the Young’s moduli of different kinds of stainless steels are almost the same. Therefore, the stiffness of the shell structure can be improved through the structure’s optimization. In view of the beam section area, the structure can be stiffness enhanced by increasing the size of the axial stiffener. Furthermore, the length of the beam can be reduced by altering the hoop stiffener to be continuous.

**Figure 15:**
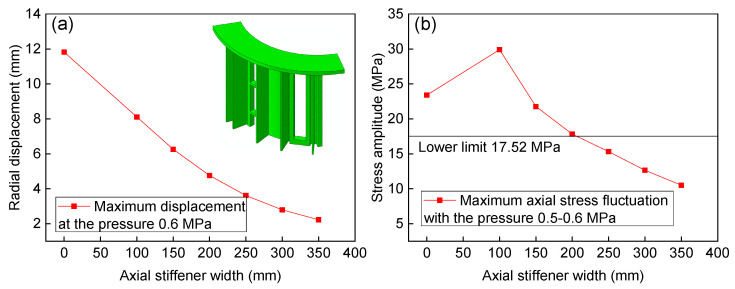
Effect of axial stiffener on the radial displacement (**a**) and fluctuating stress amplitude (**b**).

Figure 15 shows the effect of axial stiffener width on the whole structure just considering the mechanical fluctuating pressure. As the axial stiffener width increases, the section modulus in the bending of the T-shape joint increases correspondingly. The maximum radial displacement at the middle of the structure decreases linearly at the beginning, and the trend becomes slower when the axial stiffener width reaches 200 mm (see Figure 15a). Without the axial stiffener, the maximum bending normal stress amplitude in the axial direction is located at the shell structure with the magnitude 23.39 MPa. With the axial stiffener width increasing, the position of maximum axial stress amplitude transferred from the shell to the axial stiffener, and the stress fluctuating amplitude decreasing nonlinearly (see Figure 15b). The stress amplitude has reached the fatigue lower limit (17.52 MPa) when the axial stiffener width increases to 200 mm, while the radial displacement is still unsatisfied (4.76 mm). 

**Figure 16:**
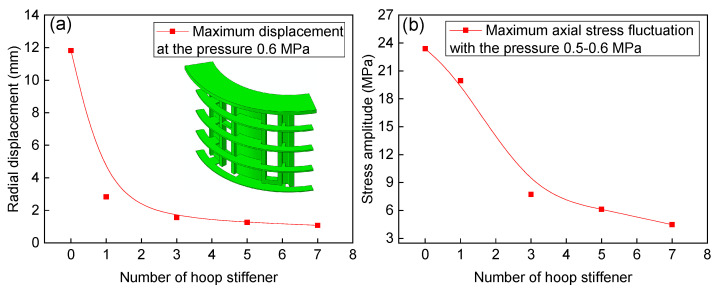
Effect of hoop stiffener on the radial displacement (**a**) and fluctuating stress amplitude (**b**).

Although a large number of stiffeners were weld to the shell structure, the stiffener in the hoop direction is discontinuous. Figure 16 shows the effect of hoop stiffener on the radial displacement and stress amplitude. The hoop stiffener has a significant effect on the radial displacement, and the radial displacement shows an exponential decrease as the number of hoop stiffeners increases (see Figure 16a). The hoop stiffener in the middle shell structure, i.e., one hoop stiffener, takes a dominant role in suppress the radial displacement. The maximum axial stress fluctuation amplitude located at the middle structure decreases correspondingly, and there is an obvious turning point at the three hoop stiffeners (see Figure 16b). In the case of three hoop stiffeners, both the radial displacement (1.563 mm) and stress amplitude (7.73 MPa) satisfy the criterion.

## 5. Conclusions

This paper presents a study on the fatigue life prediction of the shell structure of the PTA filter press using the experimental and finite element methods. By adopting the thermomechanical model and submodel approach, the accurate stress at the critical area of the stiffener is obtained. Based on the results, the conclusions are drawn as follows:

(1) The thermal load and outer structure constraints have little effect on the radial displacement and stress amplitude of the shell structure. The fluctuating pressure at the one-eighth model is the main factor affecting the displacement and stress amplitude.

(2) In the high cycle regime, both the fatigue strength reduction method and the hot spot stress method give conservative fatigue life compared with the real fatigue life. In the ultra-high cycle regime, the stress amplitude is less than the stress amplitude at the specific design fatigue life for the two fatigue design methods. The modification of the fatigue design curve of the fatigue strength reduction method gives 42% and 0.3% failure probability in the previous and present cyclic pressure conditions.

(3) The structural integrity of the shell structure is destroyed, and that part of the structure can be simplified as a T-shaped beam. Structure optimization shows that as the axial stiffener width increases, the section modulus in the bending of the T-shape joint increases correspondingly, and it subsequently decreases the radial displacement and stress amplitude nonlinearly. The hoop stiffener has a significant effect on decreasing the radial displacement and stress amplitude, and the number of three hoop stiffeners shows the most satisfactory optimization. 

## Figures and Tables

**Table 1:** Material property parameters of SAF 2205 and SS 304 [5].

Material	SAF 2205		SS 304	
Temperature (°C)	20	100	20	100
Elastic Modulus (GPa)	200	194	199	192
Poisson Ratio	0.30	0.30	0.28	0.28
Density (kg/m^3^)	7800	7800	8010	7930
Thermal Conductivity (W/m/°C)	15.00	15.00	15.26	16.30
Specific Heat (J/kg/°C)	500	500	504	523
Coefficient of Thermal Expansion (×10^−6^)	13.00	13.00	16.00	16.50

**Table 2:** Stress linearized and evaluated by design analysis.

Path	*P*_m_ (MPa)	1.5 *S*_m_ (MPa)	*P*_m_*+ P*_b_ (MPa)	3 *S*_m_ (MPa)
1	118.63	210	221.09	420
2	118.50	210	178.13	420
